# Simultaneous septic arthrodesis of the tibiotalar and subtalar joints with the Ilizarov external fixator—an analysis of 13 patients

**DOI:** 10.1007/s00590-021-03075-0

**Published:** 2021-07-29

**Authors:** Charlotte Cibura, Sebastian Lotzien, Emre Yilmaz, Hinnerk Baecker, Thomas Armin Schildhauer, Jan Gessmann

**Affiliations:** Department of Trauma Surgery and General Surgery, BG University Hospital Bergmannsheil, Ruhr University Bochum, Bürkle-de-la-Camp-Platz 1, 44789 Bochum, Germany

**Keywords:** Ilizarov fixator, Septic arthrodesis, Simultaneous arthrodesis, Subtalar joint, Tibiotalar joint

## Abstract

**Purpose:**

Treatment of joint destruction of the tibiotalar and subtalar joints caused by acute or chronic infections in compromised hosts is a challenging problem. In these cases, simultaneous septic arthrodesis with the use of the Ilizarov external fixator represents a possible alternative to amputation. This case series presents the results and complications of patients with acute or chronic infection of the tibiotalar and subtalar joints.

**Methods:**

Between 2005 and 2015, 13 patients with acute or chronic infections were treated by simultaneous single-stage debridement/arthrodesis of the tibiotalar and subtalar joints. In seven patients, there was a florid infection with fistula formation and soft tissue defects, and in six patients, there was chronic osteomyelitis with closed soft tissue. In addition to the demographic data, the time spent in the fixator, the major and minor complications and the endpoint of consolidation were reviewed.

**Results:**

The mean time spent in the fixator was 18 (min 15, max 26) weeks. The mean follow-up time for nine patients was 100 (min 3, max 341) weeks. Complete osseous consolidation of both the tibiotalar and subtalar joints was achieved in 10 patients (77%). In three (23%) patients, there was complete consolidation of one of the joints and partial consolidation of the other joint.

**Conclusion:**

The Ilizarov external fixator allows for simultaneous arthrodesis of the tibiotalar and subtalar joints in septic joint destruction. However, the healing rates are below the rates reported in the literature for isolated tibiotalar or tibiocalcaneal arthrodesis in comparable clinical situations.

## Background

Since the introduction of compression arthrodesis by Charnley in 1951, several different surgical procedures have been described as treatment options for various causes of ankle destruction [1]. Internal procedures such as the use of plates [2, 3] or screws [4] as well as intramedullary fixation [5, 6], arthroscopic fusion [6, 7] or external stabilization using external fixators can be used to achieve solid arthrodesis [1, 8–11]. However, these procedures are mainly used in aseptic patients, and the treatment of diffuse septic ankle destruction, especially in compromised hosts, remains a challenge.

Although inflammatory arthritis has been described previously [12, 13], the main causes of septic destruction are posttraumatic postoperative complications and diabetic foot syndrome [12, 14, 15]. The primary treatment goal is infection control and limb preservation with pain-free weight-bearing capacity. However, the vast majority of cases are additionally complicated by soft tissue defects, local infections, extensive scar tissue, polyneuropathy (PNP), a resistant bacterial spectrum or axial malalignments (varus/valgus). In addition, patients often suffer from other risk factors, such as alcohol or nicotine abuse, polytoxicomania, diabetes mellitus (DM) or obesity [12, 16–18]. Internal treatment options are limited in such cases [12, 19].

The Ilizarov ring fixator is an external system that provides dynamic axial fixation with high stability by introducing transfixing wires and screws outside of the infected tissue [18, 19]. In the literature, there are only a few studies on the treatment of septic arthrodesis with the Ilizarov fixator [19–22]. Most studies have a mixed aseptic/septic patient population [10, 12–16, 18, 23, 24]. Furthermore, most studies have focused on tibiotalar arthrodesis. The purpose of this study was to evaluate the use of the Ilizarov external fixator in patients with acute or chronic infections of the tibiotalar and subtalar joints.

Particular attention was given to consolidation as well as minor and major complications. Does the Ilizarov fixator achieve good results for simultaneous septic arthrodesis of the tibiotalar and subtalar joints? Are these results comparable to those described in the literature of isolated arthrodesis in only one joint?

## Patients and methods

We performed a single-center, retrospective study including all simultaneous tibiotalar and subtalar joint arthrodesis procedures performed using the Ilizarov external fixator at the authors’ institution from 01/2005 to 12/2015. To capture all patients with these criteria and their demographic data, a keyword analysis of all digitized files was performed by the author of this study (CC). The demographic data included the age and sex of the patients, associated relevant concomitant diseases, the source of the infection, time spent in the fixator, the complications and bony consolidation. The demographic data were recorded to provide a precise understanding of the complex patient population and are presented in Table 1. Following the work of Katsenis, complications were considered to be minor when conservative therapy was sufficient and major when surgical revision was required [25]. The data were collected anonymously using Microsoft Excel © Version 14.7.7. The exclusion criteria were as follows: (1) patients with isolated arthrodesis of the tibiotalar or subtalar joint, (2) tibiocalcaneal arthrodesis or (3) aseptic findings.

**Table 1:** Associated diagnosis, indication, complications, revision surgery, follow up and results (*N* = 13 Patients)

Patient	Associated diagnosis	Indication	External fixation time (weeks)	Complications (Major)	Revision surgery	Follow up and results
1	HTN	Unclear arthritic destruction with wound healing disorder and fistula formation	19	None	None	341 weeks Consolidation in the tibiotalar and subtalar joint
2	DM PNP	COM in diabetic Charcot arthropathy	12 until the major complication 18 after revision	Acute infection	Removal Ilizarov, attachment AO fixator and revision arthrodesis via Ilizarov after infection calming	142 weeks Consolidation in the subtalar joint, pseudarthrosis in the tibiotalar joint
3	None	Infection after defect filling of a talus cyst	16	None	None	17 weeks Consolidation in the tibiotalar joint, pseudarthosis in the subtalar joint
4	Osteoporosis HTN	COM after fracture	19	Instability in the midfoot	Attachment of an additional midfoot pin	3 weeks Consolidation in the tibiotalar and subtalar joint
5	None	Infectious pseudarthrosis of unknown cause (COM)	20	None	None	0 weeks Consolidation in the tibiotalar and subtalar joint
6	PNP PAOD Venous bypasses	Ankle infection in chronic plantar ulcer after injury from foreign bodies	16	2x: Break of the midfoot pin	2x: New installation of the pin	252 weeks Consolidation in the tibiotalar and subtalar joint
7	Nicotine abuse	COM after infected ligamentoplasty	16	None	None	39 weeks Consolidation in the tibiotalar and subtalar joint
8	PNP Chronic alcohol abuse HTN	COM of unclear genesis in the tibiotalar and subtalar joint	15 until first removal 26 after revision	1.: Pseudarthrosis in the subtalar joint 2.:Soft tissue defekt caused by the fixator	1.: Revision arthrodesis in the subtalar joint 2.: Remodeling of the fixator	20 weeks Consolidation in the tibiotalar joint, pseudarthrosis in the subtalar joint
9	DM Obesity HTN Nicotine abuse	Septic ankle destruction as a result of ganglion extirpation with soft tissue defect	18	None	None	0 weeks Consolidation in the tibiotalar and subtalar joint
10	DM PNP Angiopathy HTN PAOD	Septic destructed ankle after soft tissue defect with empyema in Charcot arthropathy	23	Soft tissue defekt caused by the fixator	Remodeling of the fixator	56 weeks Consolidation in the tibiotalar and subtalar joint
11	None	COM after fracture of the talus	17	None	None	33 weeks Consolidation in the tibiotalar and subtalar joint
12	HTN DM Obesity	COM after bimalleolar fracture	15	None	None	0 weeks Consolidation in the tibiotalar and subtalar joint
13	None	Acute infection with soft tissue defects after open bimalleolar fracture	18	None	None	0 weeks Consolidation in the tibiotalar and subtalar joint

*COM* chronic osteomyelitis, *DM* diabetes mellitus, *HTN* hypertension, *PAOD* peripheral arterial occlusive disease, *PNP* polyneuropathy

**Fig. 1:**
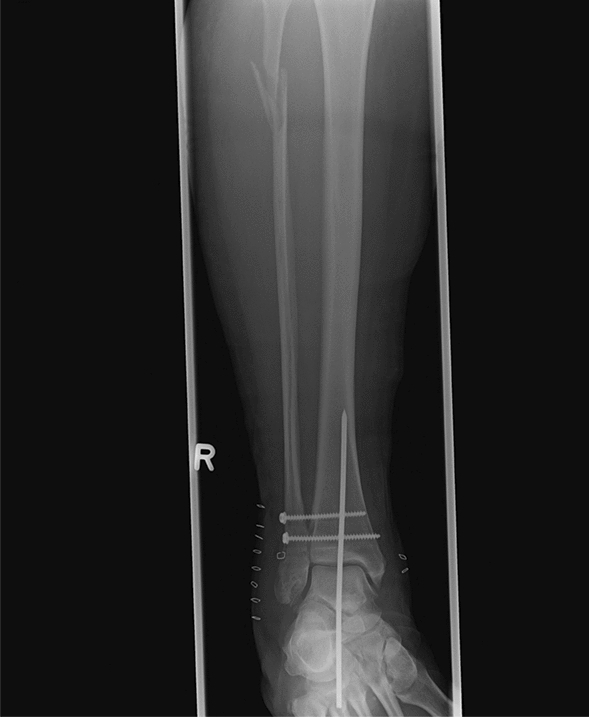
Case. A patient with a Maisonneuve fracture and secondary screw dislocation postoperatively. The initial surgical treatment as well as multiple revisions with screw replacement and K-wire osteosynthesis was performed in a different hospital

**Fig. 2:**
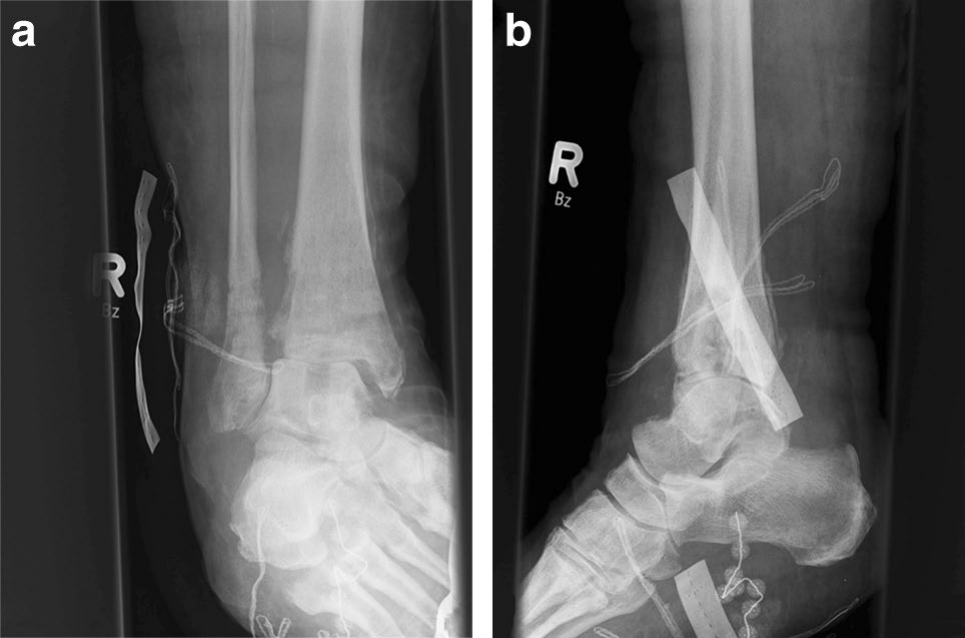
Case. **a** Image at the time of transfer to our hospital. **b** In the area of the joint, there was chronic osteomyelitis with a wound healing disorder and joint destruction

This study included a total of 13 patients (8 men and 5 women), with a mean age of 53 (min 27–max 74) years. Seven patients were classified as having chronic osteomyelitis (COM), and six patients suffered from a florid, fulminant infection with joint destruction (Case Figs. 1 and 2). All patients also showed poor soft tissue conditions characterized by pronounced scar tissue, fistula formation, soft tissue abscess and/or necrosis of the skin. The majority of patients (7/13, 54%) suffered from posttraumatic arthrosis. Other findings included Charcot arthropathies, ganglion excisions, filling of a talar cyst, and unclear arthritic destruction (Table 1). In five patients, pathogens were detected by pre- and intraoperative smears and soft tissue samples. One patient had a mixed infection of *Escherichia coli* and *Enterococcus faecalis* (Table 2). Concomitant diseases were found in eight patients, of whom six suffered from relevant diseases such as DM, PNP, alcohol abuse and/or peripheral arterial occlusive disease (PAOD). Another risk factor was nicotine abuse, which was observed in two patients.

**Table 2:** Pre- and intraoperatively proven pathogens (*N* = 6 in 5 Patients)

Pathogen	Patients
Multi-sensitive staphylococcus aureus	2
Methicillin-resistant staphylococcus aureus	1
Staphylococcus haemolyticus	1
Escherichia coli	1
Enterococcus faecalis	1

All patients were referred to us from another hospital after undergoing four operations on average (min 2–max 6). As part of the surgical treatment, all patients underwent resection arthroplasty of the tibiotalar and subtalar joints using medial and lateral approaches. Extensive debridement was performed by removing all infected cartilage, bone and soft tissue, including resection of the medial and lateral malleolus. The subtalar joint was resected by extending the lateral approach distally. The arthrodesis was positioned at 5 degrees of hindfoot valgus and 15 degrees of external rotation. Temporary fixation was carried out using 3 × 2.5 mm k-wires placed from distal to proximal through the calcaneus and tibia.

**Fig. 3:**
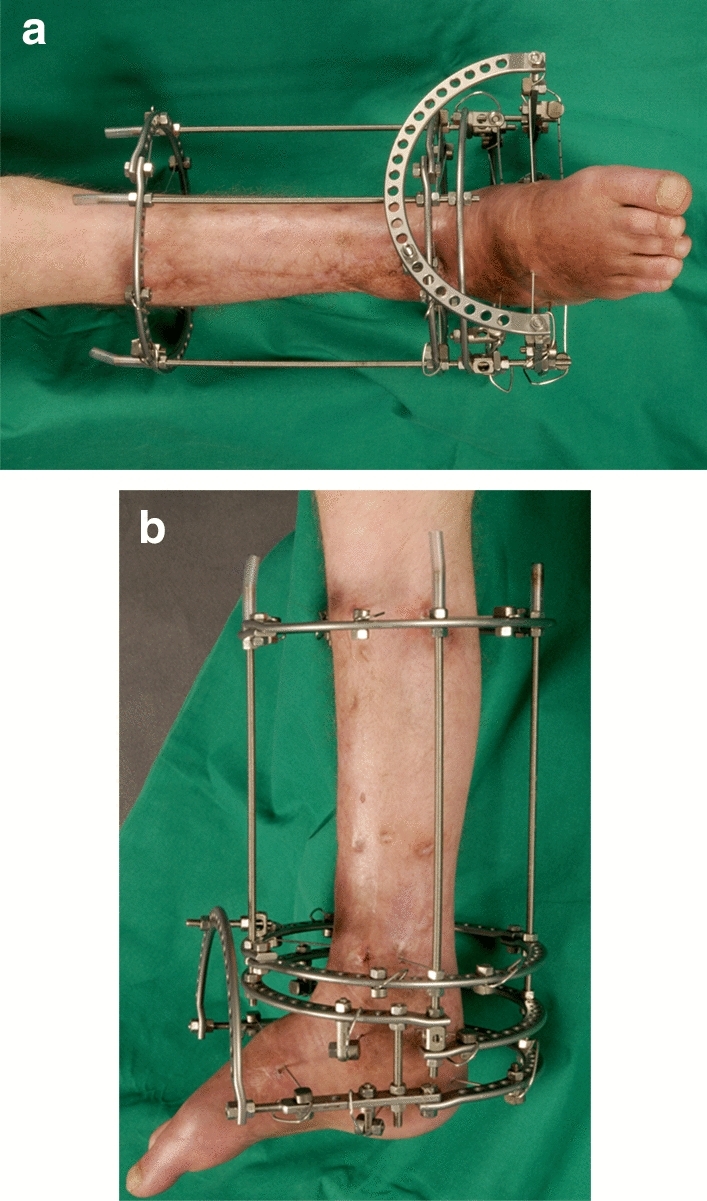
Clinical picture of an attached Ilizarov fixator for simultaneous arthrodesis

After medial and lateral wound closure, the Ilizarov frame was applied. The frames were preassembled individually preoperatively in terms of ring size and strut length and then sterilized so that a time-consuming construction did not have to be carried out intraoperatively. The frame consisted of at least four, sometimes five, ring planes, each with two crossed strained 1.8 mm steel wires per ring. The rings were placed at the level of the proximal and distal tibia, talus and calcaneus (a half-ring in the area of the calcaneus). The wires were tensioned up to 110 kg (1080 N) using the tensioning device. In addition, one or two half pins were attached for further stabilization in the area of the tibia shaft. The midfoot was transfixed with wires placed through the metatarsals (Fig. 3).

**Fig. 4:**
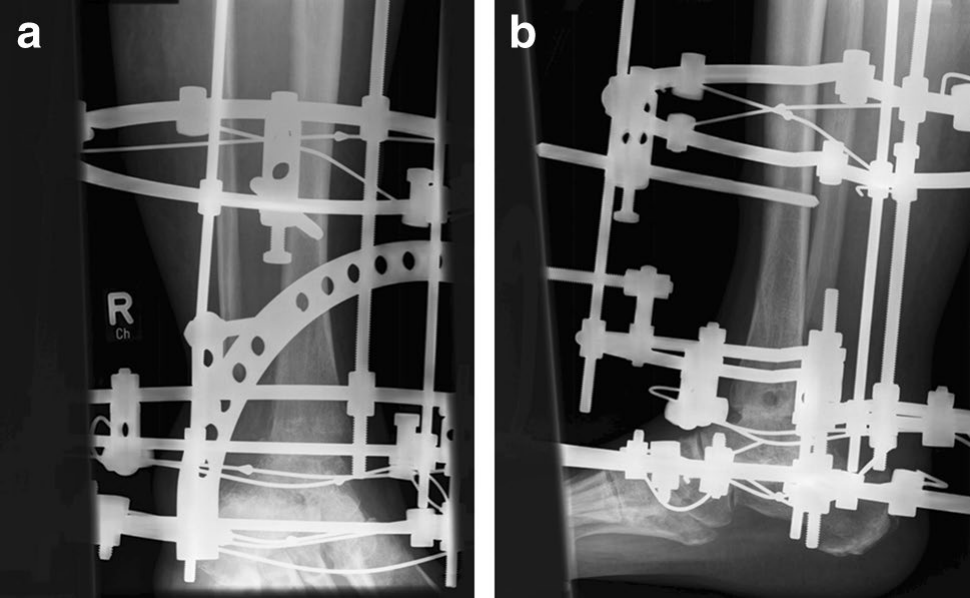
Case. **a** Image of the attached Ilizarov fixator for simultaneous arthrodesis in the tibiotalar and subtalar joints. **b** The image was taken seven weeks after surgery in our hospital

The ring system enabled compression of both the tibiotalar and subtalar joints (Case Fig. 4). In cases of soft tissue defects, a temporary wound vacuum-assisted closure (VAC) was installed, and this method was used in four patients. Two of these patients were successfully treated by this procedure with subsequent mesh graft coverage. In the other two patients, secondary wound healing was achieved by wound dressings. All patients initially received a calculated dose of antibiotics in accordance with the resistogram for a total of six weeks. The patients were allowed to perform full axial loading during the time spent wearing the fixator. To avoid the development of a pointed foot or claw toes, the patients received a forefoot plate, which could be attached to the fixator via rubber straps during both periods of rest and overnight.

After the inpatient stay, biweekly tensioning of the fixator for compression arthrodesis was applied during the consultation. (The fixator was compressed by 2 mm each time.) Radiolographical control was performed every four weeks. After removal of the fixator, the mean clinical/radiological follow-up period was 100 (min 3, max 341) weeks for a total of nine patients. Four patients were lost to further follow-up after removal of the fixator due to an invalid mailing address. Bony union was confirmed clinically and radiographically. The clinical signs included no motion at the fusion site. Union was defined radiographically using plane radiographs in four projections or computed tomography (CT) scans. In 6 patients, CT was performed to verify consolidation. Among the remaining 7 patients, there was clear consolidation on the X-ray.

## Results

**Fig. 5:**
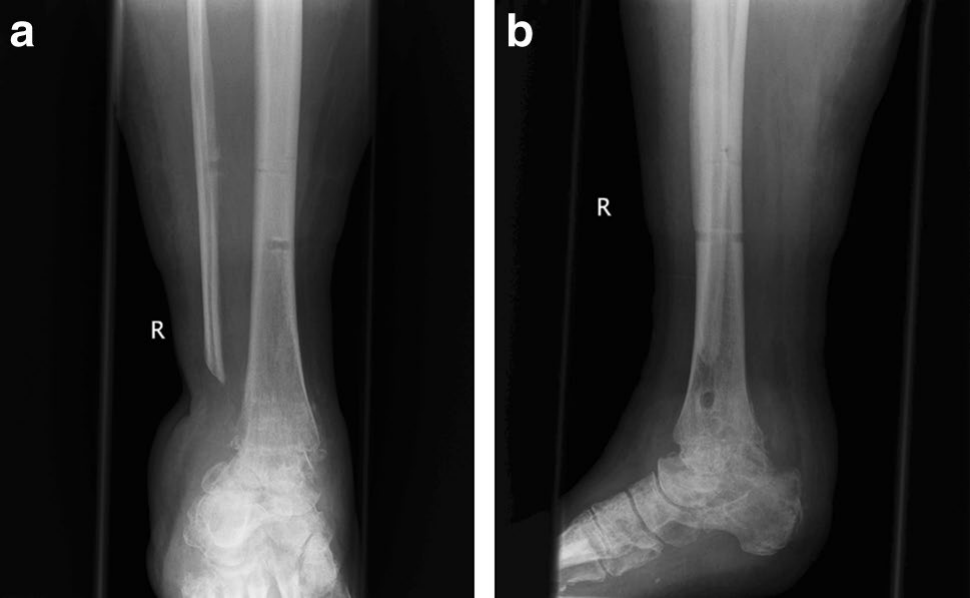
Case. **a** After 19 weeks spent in the fixator, the fixator was removed. **b **There was bony consolidation in the tibiotalar and subtalar joints

The mean time spent in the fixator was 18 (min 15–max 26) weeks. Bony arthrodesis of both the tibiotalar and subtalar joints was primarily achieved in 10 out of 13 patients (77%) (Case Fig. 5). In three (23%) patients, complete consolidation of one of the joints and partial consolidation of the other joint were found. For these patients, a conservative procedure was carried out by using carbon orthotic adaptation.

Recurrent infections did not occur during the follow-up period of a mean of 100 (min 3, max 341) weeks in nine patients. During treatment with the fixator, local pin infections occurred in all patients, but all patients could be treated locally with pin care and, if necessary, stab incision and oral antibiotics. Major procedural complications occurred in a total of seven ankles in five patients (Table 1). In patient two, a severe infection occurred after 12 weeks spent in the fixator, and removal of the fixator was necessary. Extensive debridement and installation of an AO fixator were performed. After infection control was achieved, revision arthrodesis via the Ilizarov fixator was performed (2 months after the removal of the first Ilizarov fixator). After another 18 weeks spent in the fixator, the Ilizarov fixator was removed. Consolidation of the tibiotalar joint could not be achieved, and carbon orthosis had to be adjusted. Another surgery was rejected by the patient.

In patient four, there was instability in the forefoot and an additional midfoot pin had to be inserted eight days after fixation. The time spent in the fixator was not extended, and after 19 weeks, consolidation of both ankle joints was achieved.

In patient six, a break in the midfoot pin occurred twice, and thus, a new installation was necessary. This also did not extend the time spent in the fixator, and after 16 weeks, consolidation of both joints occurred.

In patient eight, there was a soft tissue defect caused by the fixator, which is why the fixator was rebuilt (after 27 days). The fixator was removed after 15 weeks. Bony consolidation of the subtalar joint could not be achieved. Therefore, revision arthrodesis was performed, including reinstallation of the Ilizarov fixator and cancellous bone grafting. After another 26 weeks in the fixator, no complete bony consolidation of the subtalar joint could be achieved. Carbon orthosis had to be adjusted. The total duration of treatment was 60 weeks. In patient 10, there was also a soft tissue defect caused by the fixator, which is why the fixator was rebuilt. After 23 weeks, consolidation of both joints was achieved.

## Discussion

Septic arthrodesis represents a therapeutic option that could prevent limb amputation in patients with septic ankle joint destruction or COM with concomitant soft tissue defects/infections and additional risk factors such as nicotine abuse, DM or polytoxicomania [8, 13, 19, 22, 26]. However, to achieve arthrodesis in a setting of acute infection, biomechanical and biological preconditions must be met. Dynamic axial fixation with high stability against bending, shear and torsional forces, without introducing foreign material, is warranted in these cases. In addition, despite the external fixator, it is possible to treat existing soft tissue defects with, for example, VAC therapy at the same time.

The Ilizarov fixator is an available tool that exhibits all of these characteristics [10, 14, 16, 19]. By introducing transfixing wires outside of the infected tissue, stability can be achieved under poor bone and/or soft tissue conditions. With continuous compression in the area of the arthrodesis zone, which is achieved by a possible full load and by regular retightening of the fixator, bone healing is promoted [19].

Some studies of tibiotalar or tibiocalcaneal arthrodesis using the Ilizarov fixator can be found in the literature. However, the majority analysed aseptic or mixed septic/aseptic patients [10, 12–14, 16, 18, 23, 24, 27]. Healing rates in patients with acute infection or chronic osteomyelitis vary between 75 and 100%. In all of these studies, as was the case in this study, a one-step procedure was performed. Rochman et al. used a two-step approach with debridement and antibiotic chain insertion first. In five out of seven patients (71%), consolidation was reported [15]. Studies using mainly or only a septic patient population are rare [8, 19, 20, 22]. Gessmann et al. studied a population of 37 patients with septic ankle joint destruction (20 with a florid infection) and achieved primary arthrodesis in 32 patients (86.5%) [19]. Saltzmann et al. studied eight patients with COM who underwent arthrodesis using the Ilizarov fixator [22]. In 87.5% of the patients, consolidation and calming of the infection were achieved. One patient had to undergo amputation of the lower leg. Kollig et al. and Huang et al. reported 93% and 100% consolidation rates, respectively. However, they used a hybrid fixator, not a ring fixator [8, 20]. Although a hybrid fixator is not as bulky or expansive as an Ilizarov ring fixator, it has the disadvantage that a full axial load cannot be applied; thus, early mobilization cannot be achieved. Therefore, complete load removal of the leg during the healing period under constant thrombosis prophylaxis is necessary.

The success rate of 77% for the septic patient population found in our study is lower than the rates found in the cited studies. However, in our study, with similar patients, the tibiotalar and subtalar joints were simultaneously addressed. Only Saltzman et al. reported simultaneous treatment of both joints in four septic patients, and simultaneous bony arthrodesis was reported in three out of four patients. In all of the other studies mentioned, either tibiotalar or tibiocalcaneal arthrodesis was performed [10, 12–16, 18, 19, 23, 24, 27].

The rate of major procedural complications of 46% also appeared to be relatively high in our study, although the data in the literature vary regarding this rate. Gessmann et al. reported a major complication rate of 16% with three pin breakages and three deep pin infections. Deep pin infections did not occur in our patients, but breakage of the pin did occur in two patients. Saltzmann et al. also reported a pin break. However, in a total of eight patients, a pin break was the only major complication described. A tibial pin was reported to be broken [22]. Johnson et al. reported the need for premature fixator removal due to intolerance of the fixator. No other major complications were reported in a total of six patients [10]. Fragomen et al., Salem et al. and Zarutsky et al. reported a similar incidence of major procedural complication rates in mixed septic/aseptic patients, with 32%, 36%, and 51% complication rates, respectively, compared to the rate found in our study, although it was not a purely septic patient population. However, the time spent in the fixator was longer than in other studies, an average of 25 weeks (Fragomen et al.) and 27.7 weeks (Salem et al.), and in our study (18 weeks), which can be explained by simultaneous segmental transport of the fixator [12, 16].

Other complications have been reported, including tibial stress fractures, pseudarthroses, pseudarthroses with deformities greater than 10 degrees, fulminant infections, tibial nerve neurapraxia and necrosis of the talus [12, 16]. In addition to pin breaks and forefoot instability, our study also found fixator-related soft tissue defects (2x), pseudarthrosis (1x) and one fulminant infection. Zarutsky et al. reported that major complications occurred more frequently in patients with side effects such as DM, Charcot arthropathy, or septic fusion [18]. Other studies also described an increased complication rate in patients with difficult soft tissue conditions, circulatory disorders, and nicotine and alcohol abuse [12, 28, 29]. This result was confirmed in our study. Four out of the five patients with major complications had DM, PAOD, Charcot arthropathy, nicotine consumption and/or alcohol abuse.

This study had several limitations. The study sample of 13 patients was small, and the study had a retrospective design. Furthermore, a large number of patients were lost to follow-up. We treat patients from all over the country, which is why regular follow-up is not always possible.

Due to the small number of patients, this work cannot be the basis of a standardized therapy recommendation. This would require further studies with a larger patient population. In addition, comparable studies with simultaneous arthrodesis of the tibiotalar and subtalar joints are not available. There was also no long-term clinical follow-up for the evaluation of infection-free patients or subjective patient satisfaction. However, the results presented here show that this method represents an alternative to amputation for selected patients. However, clinical decisions will require a detailed explanation of the long and complex treatment times as well as the procedural complications on a case-by-case basis.

## Conclusion

The Ilizarov fixator allows for simultaneous arthrodesis of the tibiotalar and subtalar joints in septic joint destruction. However, the complication rates of this procedure are high, and the healing rates are lower than the rates for isolated tibiotalar or tibiocalcaneal arthrodesis in comparable clinical situations, as described in the literature. More research with a larger patient sample needs to be performed to confirm our results.

## Data Availability

All of the data are electronically available.

## Abbreviations

CT: Computed tomography
COM: Chronic osteomyelitis
DM: Diabetes mellitus
HTN: Hypertension
PAOD: Peripheral arterial occlusive disease
PNP: Polyneuropathy
VAC: Vacuum-assisted closure
